# Hybrid Materials Based on Nanoparticles Functionalized with Alkylsilanes Covalently Anchored to Epoxy Matrices

**DOI:** 10.3390/polym14081579

**Published:** 2022-04-13

**Authors:** Alexis Salas, Andrés Felipe Jaramillo, Daniel Andrés Palacio, Andrés Díaz-Gómez, David Rojas, Carlos Medina, Eduardo Pérez-Tijerina, Francisco Solís-Pomar, Manuel Francisco Meléndrez

**Affiliations:** 1 Department of Mechanical Engineering (DIM), Faculty of Engineering, University of Concepción, Edmundo Larenas 219, Concepcion 4070409, Chile; asalasmec@gmail.com (A.S.); cmedinam@udec.cl (C.M.); 2 Interdisciplinary Group of Applied Nanotechnology (GINA), Hybrid Materials Laboratory (HML), Department of Materials Engineering (DIMAT), Faculty of Engineering, University of Concepcion, 270 Edmundo Larenas, Box 160-C, Concepcion 4070409, Chile; andresdiaz.qind@gmail.com (A.D.-G.); davrojas@udec.cl (D.R.); 3 Department of Mechanical Engineering, Universidad de La Frontera, 01145 Francisco Salazar, Temuco 4780000, Chile; andresfelipe.jaramillo@ufrontera.cl or; 4 Mechanical Engineering Program, School of Engineering, Universidad Tecnológica de Bolívar, Parque Industrial y Tecnológico Carlos Vélez Pombo km 1 Vía Turbaco, Cartagena de Indias 130001, Colombia; 5 Department of Analytical and Inorganic Chemistry, Faculty of Chemistry, University of Concepción, 129 Edmundo Larenas, Concepcion 4070409, Chile; dapalacio@udec.cl; 6 Facultad de Ciencias Físico-Matemáticas, Universidad Autónoma de Nuevo León, San Nicolas de los Garza 66451, Mexico; eduardo.pereztj@uanl.edu.mx (E.P.-T.); francisco.solispm@uanl.edu.mx (F.S.-P.); 7 Unidad de Desarrollo Tecnológico, Universidad de Concepcíon2634 Av. Cordillera, Parque Industrial Coronel, Box 4051, Concepción 4191996, Chile

**Keywords:** alkylsilanes, epoxy resin, ZnO, hybrid materials, nanocomposite, functionalization

## Abstract

In this work, the surface modification of zinc oxide nanoparticles (ZnO-NPs) with 3-glycidyloxy-propyl-trimethoxysilane (GPTMS) was investigated. The ZnO-NPs were synthesized using the physical method of continuous arc discharge in controlled atmosphere (DARC-AC). The surface modification was carried out using a chemical method with constant agitation for 24 h at room temperature. This surface functionalization of zinc oxide nanoparticles (ZnO-NPs-GPTMS) was experimentally confirmed by infrared spectroscopy (FT-IR), TGA, and XRD, and its morphological characterization was performed with SEM. The increase in mechanical bending properties in the two final hybrid materials compared to the base polymers was verified. An average increase of 67% was achieved with a moderate decrease in ductility. In the case of compressive strength, they showed mixed results, maintaining the properties. With respect to thermal properties, it was observed that inorganic reinforcement conferred resistance to degradation on the base material, giving a greater resistance to high temperatures.

## 1. Introduction

The development of organic–inorganic hybrid materials, which is often achieved by grafting synthetic polymers onto inorganic particles or by the addition of modified nanoparticles (NPs) to polymeric matrices, is intended to produce composite materials with improved mechanical and other properties. Inorganic nanoparticles and organic polymer nanocomposites represent a new class of materials that exhibit improved performance compared to their microparticle counterparts [1]. Surface modification of inorganic nanoparticles has attracted considerable attention because it produces excellent integration and improved interface between nanoparticles and polymer arrays [1,2], which in turn can be used in various of applications in different fields such as medicine, textiles, electronics, environment, food packaging, and paints [1,2,3,4,5].

Surface modification of metal oxides with alkylsilanes has become one of the most widely used methods for preparing a monolayer of the products of the coupling reactions. This is achieved with OH-bearing surfaces, which have uses or applications in various areas [6]. The main advantage is the rapid formation of covalent bonds between the substrate and the anchoring groups. This covalent bond allows the stabilization of the monolayer and facilitates further chemical modification without compromising the integrity of the surface film being formed. Related research has been conducted on the properties of these films, i.e., the chemical composition, thickness, orientation, and lateral order of alkyl chains on a wide range of surfaces, such as zinc oxide [7], iron oxide [8], and silica [9].

Polymer arrays reinforced with modified inorganic nanoparticles combine the functionalities of polymer arrays, which include low weight and easy conformability, with the unique characteristics of inorganic nanoparticles [10]. Nanocomposites obtained by the incorporation of this type of material can lead to improvements in several areas, such as optical, mechanical, electrical, magnetic, rheological, and fire-retardant properties. However, nanoparticles have a strong tendency to become agglomerated followed by insufficient dispersion in the polymer matrix, degrading the optical and mechanical properties of nanocomposites [11,12]. To improve the dispersion stability of nanoparticles in aqueous media or polymeric matrices, it is essential that the surface modification of particles involving polymer surfactant molecules or other modifiers generates a strong repulsion between nanoparticles. An additional problem found in nanocomposites is a lower impact resistance than that found in the organic precursor only due to the stiffness of the inorganic material, leading to the use of additives to increase the tenacity of the compounds [10,13]. There are many studies on the implementation of NPs in polymers to increase their functionality and improve the dimensional stabilization of nanocomposite materials in medical, optical, and coating applications [14]. Some authors have reported on the positive effects of surface-modified oxide nanoparticles on the mechanical properties of nanocomposites [15]. Among these modification techniques, silane coupling agents have gained more attention for their bifunctional structures. For example, H. Amirbeygi et al. [16] studied the surface modification of graphene nanoplates by introducing aminopropyl trimethoxysilane, and they also evaluated the effect of the modification on the tensile, compression, interlaminar shear stress (ILSS), and tribological properties of a nanocomposite of epoxy matrix, obtaining significant improvements in each of these properties. A very similar work was carried out by A. Mostovoy and collaborators [17] who modified carbon nanotubes with γ-aminopropyltriethoxysilane (APTES) and dispersed within an epoxy matrix. The results showed that the functionalization and chemical compatibility of the carbon nanotubes treated with APTES provides an increase in the physico-mechanical properties of the nanocomposite material.

Note that the morphology of nanoparticles, dispersion, size, and concentration should be taken into account when adding nanostructures to the polymer matrix, the distribution being one of the most important factors to be achieved at the experimental level due to the possible or high trends of nanostructured materials to form aggregations when obtaining hybrid materials or nanocomposite [18,19,20]. However, some investigations have obtained homogeneities in the distribution of the NPs in the matrix and in turn their dispersion, which helps control, entropically or enthalpically, the interface between the matrix and the nanoparticles [1,20]. Kumar et al. [21] presented strategies that can improve the distribution of nanoparticles and minimize the enthalpic contribution; the first is to increase the interactions between the matrix and NPs through silane coupling agents [18,19,20] and other low molecular weight molecules, and the second is the use of high molecular weight polymers grafted onto the surface of the NPs that are distributed in the matrix [1].

On the other hand, different methods for obtaining metal oxide nanoparticles have been studied [22,23], where a disadvantage of these techniques is obtaining NPs on a small scale [24,25]. In this research, zinc oxide nanoparticles (ZnO-NPs) were obtained, important semiconductor materials that have attracted the attention of the scientific community for their good optical, photonic, and electronic properties with applications in many fields. They are characterized by their nontoxicity, biocompatibility, and high sensitivity to different environmental conditions [2,26,27]. These nanoparticles were obtained using the continuous arc discharge technique in a controlled atmosphere (DARC-AC), which is a highly promising technique due to its great versatility and low cost. Hybrid materials were then obtained in conjunction with a polymer matrix based on epoxy resins.

Different studies have been reported in literature concerning the addition of nanoparticles in epoxy resins, such as Fe_3_O_4_/rGO nanoparticles, to improve abrasion resistance for adsorbent coatings [28], Prabhu et al. [29] evaluated mechanical and gamma-ray protection properties using Ta_2_O_5_ micro and nanoparticles in epoxy resins, Chen et al. [30] added multi-walled carbon nanotubes to epoxy resins. Furthermore, the addition of ZnO nanoparticles, such as in the study of Thipperudrappa et al. [31], has been specifically reported in studying the influence of ZnO-NPs on the mechanical and thermal responses of epoxy resin nanocomposites, demonstrating that the addition of nanoparticles improves mechanical properties such as tensile and bending strength due to affinity with the matrix. Wang et al. [32] studied the dissipation of surface loads in gas insulation system insulators using ZnO/epoxy NPs. They demonstrated that the increase in the concentration of NPs promotes the dissipation of the surface load, in line with other reported works [33,34,35,36]. This research work was based on the production of hybrid materials from nanoparticles of metal oxides (ZnO) obtained by DARC-AC and functionalized by 3-glycidyloxy-propyl-trimethoxysilane (GPTMS) as filling agents bound by covalent interactions in epoxy matrices. The coupling reaction was verified by FT-IR, and morphological and thermomechanical characterizations of the hybridized developed material were performed.

## 2. Materials and Methods

The raw materials used for the synthesis of ZnO-NPs by the controlled atmosphere arc discharge method (DARC-AC) were oxygen and Zn wires of 2 mm diameter of high purity (99.99%) (Sulzer, Winterthur, Switzerland). For the functionalization of the ZnO-NPs, 3-glycidyloxy-propyl-trimethoxysilane (GPTMS) (Sigma-Aldrich, Burlington, MA, USA), toluene and ethanol (Sigma-Aldrich, Burlington, MA, USA) were used. The methodology of synthesis and functionalization, in addition to the structural and morphological characterization and monitoring of the coupling reaction, was developed in accordance with previous studies carried out by our team [37]. Two types of epoxy resin (R1 and R2) were used together with their respective hardeners: epoxy l20-bisphenol A (R1) cross-linked with hardener EPH 573 (H1) and bisphenol F (R2) cross-linked with HT2 (H2) (R&G Faserverbundwerkstoffe GmbH, Waldenbuch, Germany). The composition of the resins and hardeners used is shown in Table 1.

### 2.1. Nanoparticle Synthesis of ZnO by Controlled Atmospheric Arc Discharge (DARC-AC)

**Figure 1:**
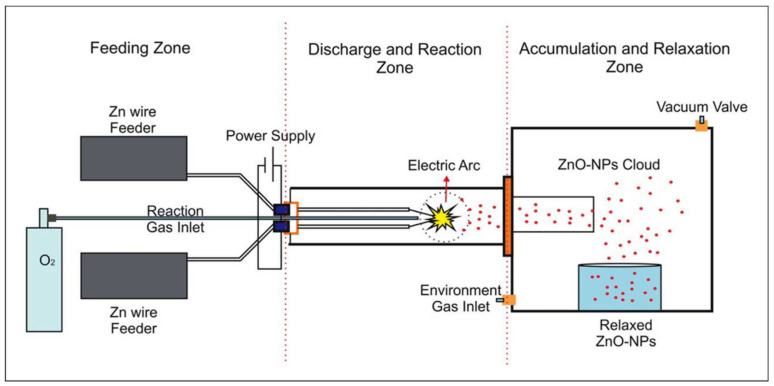
Schematic of equipment based on controlled atmosphere arc discharge (DARC-AC1), used for ZnO nanoparticle synthesis, without passivated surface.

The synthesis was carried out using a high-purity Zn wire (99.99%) in the presence of oxygen as a carrier and oxidizing gas. The typical reaction to produce ZnO-NPs comprises an O_2_ flow of 50 sccm, a precursor wire velocity of 1.5 cm/s, electrode contact angle of 45°, and reaction voltage set at 220 V. The reaction was pulsed to prevent reactor overheating, with nucleation time, growth, and relaxation of 60 min. Nanoparticles were collected in a storage chamber in an inert atmosphere. A more detailed schematic of the controlled atmosphere arc discharge machine (DARC-AC) can be found in Figure 1 [38].

### 2.2. Surface Modification of ZnO Nanoparticles Using GPTMS

The reaction was performed by coupling the silane reactive groups on the surface of the ZnO-NPs by incorporating bidentate molecules of GPTMS in the presence of toluene. For the modification process, 6.0 g of ZnO-NP powder and 180.0 g of toluene were added to a round-bottomed flask with constant stirring at ambient temperature. After 5 min, the suspension was treated in an ultrasonic bath for 10 min, and then 3 g of GPTMS was added to the mixture, maintaining constant agitation. The homogeneous mixture obtained was subjected to continuous agitation for 24 h at room temperature. ZnO powders modified with GPTMS were subjected to three ethanol washes to remove the molecules of GPTMS without reacting. The powders were dried at 80 °C for 12 h [37]. The functionalized powders obtained were labeled ZnO-NPs-GPTMS.

### 2.3. Production of Hybrid and Nanocomposite Material

The hybridized material and nanocomposite material were produced by mixing the two types of resin with their respective hardeners in the proportions suggested by the data sheet in a 4:1 ratio for the combination R1/H1 and 2:1 for the mixture R2/H2. Once the mixture was obtained, it was deposited in Petri dishes and in plastic containers to be stirred for a period of 10–15 min. The modified and unmodified ZnO-NPs were added to the mixtures in the proportions shown in Table 2. To obtain the material, four methods of agitation were used: manual (glass rod), mechanical (vortex), a combination (vortex + glass beads), and ultrasound. After stirring, the mixture was deposited in silicone molds with dimensions per test piece for subsequent analysis and then left to cure for approximately 24 h in the oven at room temperature (Figure S1, Supplementary Material). The nanocomposite materials obtained with R1 and R2 were labeled NCM1 and NCM2, while the hybrid materials were labeled as HMF1 and HMF2, respectively.

### 2.4. Characterization

#### 2.4.1. Morphological and Structural Properties

A structural analysis was performed using XRD and a morphological analysis using TEM and SEM. TEM micrographs of ZnO modified and unmodified powders were obtained using a JEM 1200 EX II transmission electron microscope (JEOL, Ltd., Tokyo, Japan) at a voltage of 120 kV. The sample was prepared by placing a drop of nanoparticles diluted in ethanol on a 200-mesh carbon-coated copper grid. XRD spectra of the modified and unmodified powders were obtained using a Bruker endeavor diffractometer (model D4/MAX-B; Bruker, Billerica, MA, U.S. model), with a sweep of 4.0 to 80.0° 2θ with a step of 0.02° and a counting time of 1 s. The diffractometer was operated at 20 mA and 40 kV with a copper cathode lamp (λ = 1.541 Å).

Using scanning electron microscopy (SEM), the morphology of hybrid and nanocomposite materials could be observed. This analysis was performed using a scanning electron microscope (Jeol Model JSM 6300 LY) with 20 kV acceleration voltage. Samples previously immersed in liquid nitrogen were coated by sputtering with gold approximately 50 nm thick. The samples were also analyzed by dispersive energy X-ray (EDS) spectroscopy. Finally, to detail the polymerization reaction of hybrid and nanocomposite materials and functionalization reaction in ZnO-NP powders with silane molecules, an FTIR characterization was performed. The spectra were obtained using an FTIR Spectrum Two spectrometer (x 1720X) (Perkin Elmer, Waltham, MA, USA) with the total attenuated reflection function (ATR). Each spectrum was obtained by consecutive scans in the range of 4000 to 500 cm^−1^ with a resolution of 1 cm^−1^.

#### 2.4.2. Mechanical Properties

To evaluate the effect of adding NPs on the mechanical properties of the materials obtained, three-point bending and compression tests were performed, following the parameters established in the ASTM-D790 and ASTM-D695 standards, respectively. The tests were performed on a smarTens universal test machine (model 005; Emmeram Karg Industrietechnik, Krailling, Germany) with a 1 kN load cell. The test pieces were produced in silicone molds with dimensions of 60 × 2.9 × 10 mm for bending tests and 10 × ø 5.5 mm for compression tests. For the bending tests, three parameters were evaluated: bending modulus (E_f_), bending resistance (σ_fm_), and bending strength deformation (ε_fm_); meanwhile, for the compression tests, three parameters were evaluated: compression modulus (E_c_), compression resistance (σ_cm_), and compression deformation (ε_cm_).

#### 2.4.3. Thermal Properties

The TGA analysis was performed using a thermo-microbalance TG 209 FIIris^®^ (NETZSCH-Gerätebau GmbH, Selb, Germany) with an aluminum sample holder. The mass change as a function of temperature was measured using a heating ramp of 20.0 to 600 °C at a speed of 10 °C/min under an N_2_ atmosphere.

## 3. Results and Discussion

### 3.1. Synthesis of ZnO Nanoparticles by DARC-AC

**Figure 2:**
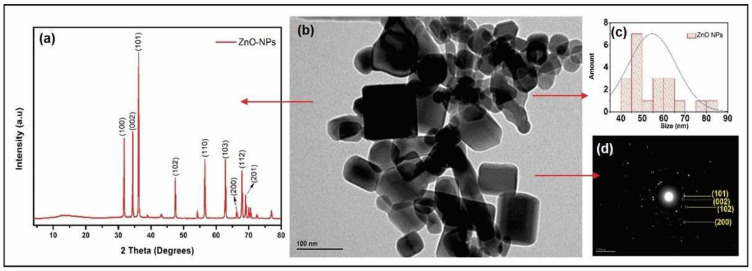
(**a**) XRD diffractogram of ZnO nanoparticles synthesized using DARC-AC1, (**b**) TEM micrography of obtained nanoparticles, (**c**) frequency histogram, and (**d**) SAED of ZnO-NPs.

In order to determine the morphology of the nanostructures, XRD and TEM analyses were performed. In Figure 2a, the DRX pattern is shown, where characteristic peaks at 2θ = 31.7°, 33.9°, 36.2°, 47.3°, 56.4°, 62.7°, 67.8°, and 68.8° correspond to planes (100), (002), (101), (102), (1110), (103), (200), (112), and (201), respectively, which can be indexed to a wurtzite-type hexagonal ZnO material. Similar results are reported in the research of C. Medina et al. [38], who performed the synthesis of zinc oxide nanoparticles through the same method, showing characteristic peaks of zinc oxide nanoparticles; this corroborates the production of high purity wurtzite zinc oxide. These results are in agreement with those shown in Figure 2d, where interplanar distances (d_hkl_) of 2.60, 2.48, 1.91, and 1.14 Å were shown, corresponding to planes (002), (101), (102), and (202) for ZnO hexagonal-type wurtzite.

In the TEM micrographs (Figure 2b), a heterogeneous distribution of the ZnO-NPs is observed, while their morphology is distanced from a spherical form, cataloged with a polyhedral-type morphology. The histogram associated with these measurements is shown in Figure 2c, and the size of nanoparticles ranged between 45 and 60 nm. These results are consistent and close to those reported by A. F. Jaramillo, et al. [37]. That is to say that the final size depends on the synthesis procedure. For the DARC-AC method, it can be achieved by varying the contact angle, the cable speed, or the gas flow, leading to nanoparticles with a smaller or larger size according to the established parameters [37,38].

### 3.2. Surface Modification of ZnO Nanoparticles by GPTMS

**Figure 3:**
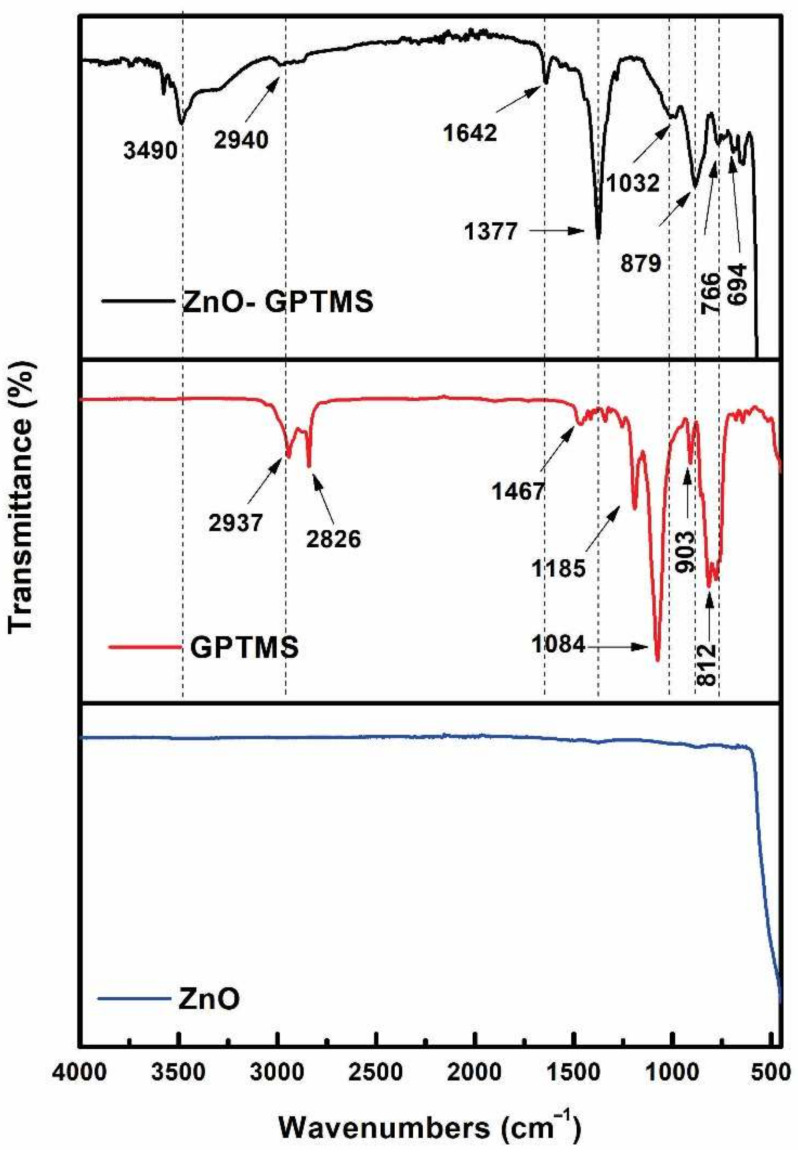
FTIR, ZnO-NPs, GPTMS, and ZnO-NPs-GPTMS spectra.

Infrared spectroscopy (FT-IR) analyses for nonfunctionalized and functionalized nanoparticles with GPTMS were performed to determine the process of functionalization of the NPs. In Figure 3, the presence of different bands between the ZnO-NPs and the nanoparticles modified by the silane coupling agent is shown. These results are in complete agreement with those found by R. Moussawi [39]. For the ZnO-NPs-GPTMS, peaks are exhibited at 698 and 904 cm^−1^, which are attributed to Si-O bonds. Similarly, at 1030 cm^−1^ a small band attributed to vibrations is observed in the Si-O-Si bond formation. Finally, at 2940 cm^−1^ the absorption band belonging to the vibration C-NH is presented. These results are comparable and consistent with those reported by A. Amaria [40] who studied the modification of SiO_2_ particles using GPTMS to coat Fe_2_O_4_ nanoparticles. It is well known that silane coupling agents are first hydrolyzed to silanol, and then condensation reactions occur between silanes and the hydroxyl groups of the substrate surface. However, there is a special interaction between the amino group and the ZnO surface. Note that there are different types of interactions between the amino silane group and the ZnO surface reported in our previous work [37]. The first is that the amino group can present hydrogen bond interactions with the hydroxyl groups present on the surface of the NPs, and the second mechanism corresponds to a covalent bond condensation between the oxygen and the surface of the ZnO-NPs. In Figure S2, the mode of functionalization of the NPs with the GPTMS molecules is proposed due to the steric impediment that yields the inference of a condensation mechanism.

### 3.3. Production of Hybrid and Nanocomposite Material

#### Polymerization Reaction Study

It is important to clarify that hybrid materials based only on hardeners and GPTMS (HM1-G and HM2-G) were mainly developed to verify whether there was a noticeable change in the viscosity of the mixture; in these cases, no resin was used because the aim was to test if the functional group epoxy of the alkylsilane reacted with the diamine (hardener). If this change existed, this meant that some degree of reaction was achieved between the hardener and GPTMS. In this case it is impossible to speak of cross-linking because the alkyl silane has only one epoxy group; therefore, changes in the rheology of the mixture could provide information about the reaction. Then, we proceeded with the synthesis of a final hybrid material by mixing all the components and adding functionalized and functionalized ZnO-NPs. In the case of HM1-G and HM2-G materials, a change in the viscosity of the mixture was observed in the previous polymerization studies, even though a constituent was not present (the resin). The answer to the previous phenomenon lies in the concept of reactivity. The reactivity of a group depends mainly on three factors: its chemical atmosphere, i.e., what occurs chemically around it; the functional groups present and the steric effect, in other words, large groups present and have a particular influence on the chemical reaction [9,41,42].

**Figure 4:**
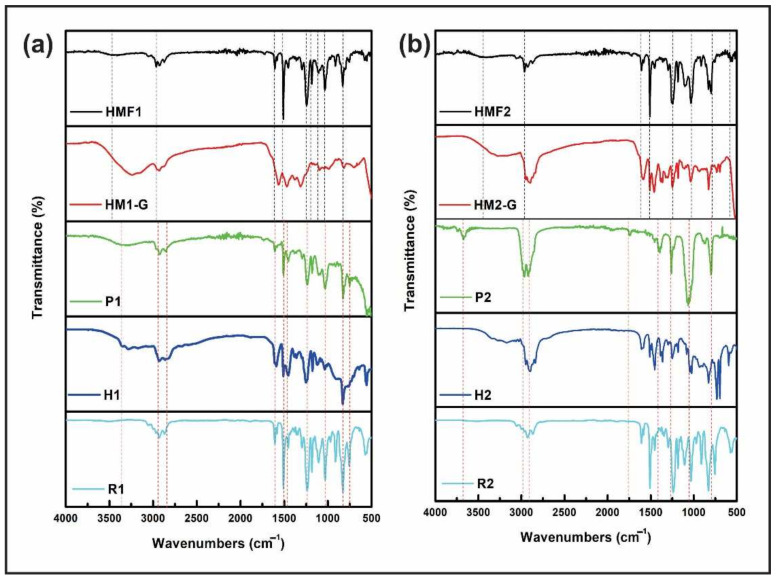
Comparison of FTIR spectra of the hybrid materials obtained (HMF1 and HMF2), obtained with the two resins (**a**) R1 and (**b**) R2, with their respective hardeners. The spectrum of the single cross-linked resin (P1 and P2) and the resin mixed with the GPTMS is presented to verify reactivity.

The FT-IR spectrum for the cross-linked polymer (P1) produced from the mixture of R1 and H1 is shown in Figure 4a. A peak at 1261 cm^−1^ can be indexed to the movement of the C-N bond that is formed when the material intersects; in addition, the vibration N-H at 880 cm^−1^ was appreciated. Making a comparison of each component with P1, a peak at ~880 cm^−1^ is detailed for the case of the hardener that corresponds to the vibration N-H and which coincides with that found in the polymer P1 in the spectrum. In the same way for the case of the resin, the bond C-O at 1100 cm^−1^ is visualized. This bond is not verified in the spectrum of the polymerized material. Conducting the same analysis for the R2 and H2, the spectra of which are shown in Figure 4b, a peak at 1081 cm^−1^ is exhibited, which can be associated with the vibrational movement of the C-N bond. For the case of the hardener E2, a band at 714 cm^−1^ corresponding to the N-bond is visualized for the material. Finally, for the R2 resin a bond is verified at 1122 cm^−1^, which corresponds to the vibration of the C-O bond. Thus, according to the literature [43], the polymerization process was performed well.

**Figure 5:**
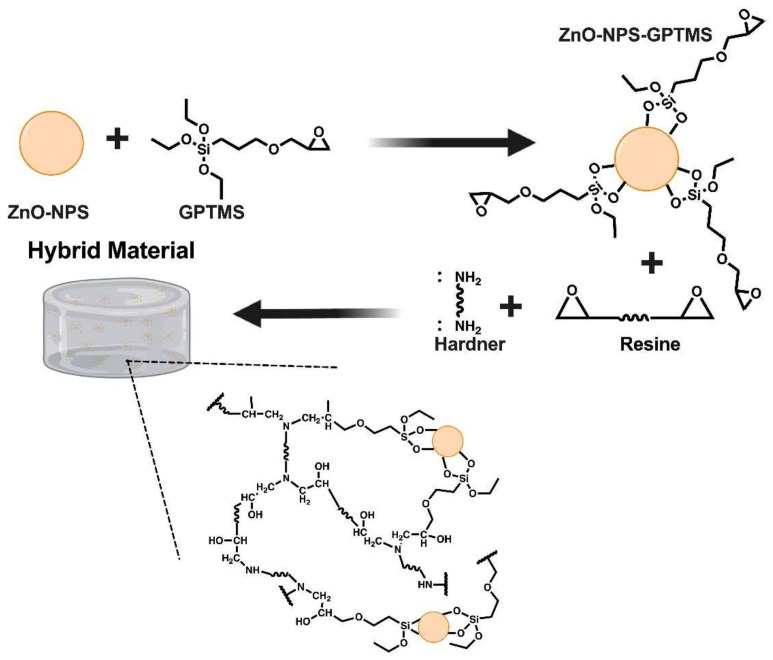
The invention relates to a mechanism for the synthesis of hybrid material by combining the epoxy resin and the ZnO-NPs-GPTMS to obtain the three-dimensional cross-linked network.

In addition, an FT-IR analysis was performed on the final hybrid material manufactured with the combination of the R1 resin with the E1 hardener and functionalized ZnO nanoparticles (ZnO-NPs-GPTMS) to verify the changes compared to the P1 polymer. A comparison between P1 and the final hybrid material HMF1 is shown in Figure 4a. As you can see, there are certain differences in terms of vibrational bands referred to between the P1 polymer. We can see that, in the polymer P1 at ~1080 cm^−1^, a peak belonging to the vibration C-O is visualized. This peak in the hybrid material is split; one remains at 1030 cm^−1^, and another, at ~1100 cm^−1^. This splitting indicates that two chemical reactions are occurring: one between the NH2 group of the hardener and the ZnO-NPs-GPTMS and one between the other NH2 group of the hardener and the epoxy resin (see Figure 5). At approximately ~1030 cm^−1^ is the Si-O-Si band. This peak in the final hybrid material HMF1 corresponds to the junction between the NH2 group and functionalized nanoparticles, while the one on its left exhibits the resin-hardening anchorage. On the other hand, in the spectrum belonging to HMF1 there is a band at 1184 cm^−1^, which can be associated with the vibrational movement of the C-N bond. The polymerization occurs when the chain breaks, passing from a C-O bond to a C-N bond. The band at 1184 cm^−1^ demonstrates the bonding of the ZnO-NPs-GPTMS epoxy group to the hardener, breaking to form the bonding. Additionally, at 3414 cm^−1^ there is a band that can be associated with the SiO-H vibrational movement, which is typical of functionalization. The results of the vibrations obtained are consistent with the study of S. Suresh [44], who worked with the GPTMS silane and found the proper bands of the functionalization process, called the anchor between silicon and oxygen.

Meanwhile, for the hybrid material HMF2, the spectra shown in Figure 4b exhibits similar changes, as far as vibrational movements are concerned. That is, at 1292 cm^−1^, a vibration movement is shown that can be attached to a C-N bond; the split of the peak is verified to the ~1080 cm^−1^ belonging to the polymer P2 in two new peaks: one at 1028 cm^−1^ and another at 1105 cm^−1^. The first is associated with the anchoring of the ZnO-NPs, since it represents the Si-O-Si bond, and the second to a C-O bond, typical of the union between the resin and the hardener. The SiO-H vibration is also displayed at 3435 cm^−1^, from the same functionalization process. Spectra were made of the polymer obtained with each resin (P1 and P2), which were compared with the polymers obtained by mixing the resin, hardener, and GPTMS (HM1-G and HM2-G), and the hybrid material obtained by the cross-linking reaction of each resin with its respective hardener and the ZnO-NPs-GPTMS (HMF1 and HMF2), in order to identify the characteristic signals of each material and to be able to ensure the formation of the hybrid material. The synthesis mechanism for HMFs is shown in Figure 5.

### 3.4. Mechanical Properties

**Figure 6:**
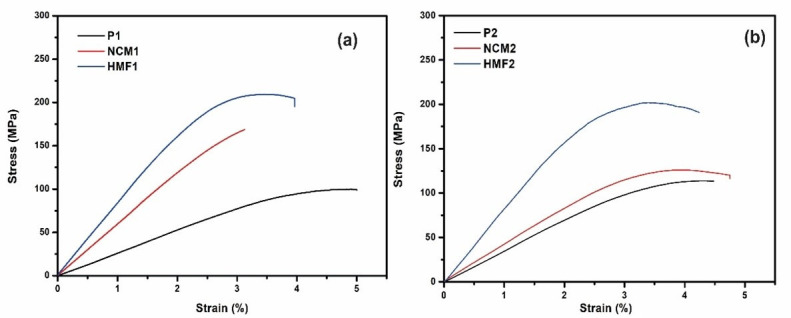
Graphic of the bending resistance for (**a**) test pieces made with cured resin 1 (P1), nanocomposite made with resin 1 and ZnO-NPs without functionalization (NCM1), and hybrid material (HMF1) and (**b**) test pieces made from cured resin 2 (P2), nanocomposite made from non-functioning resin 2 and ZnO-NPs (NCM2), and hybrid material (HMF2).

**Figure 7:**
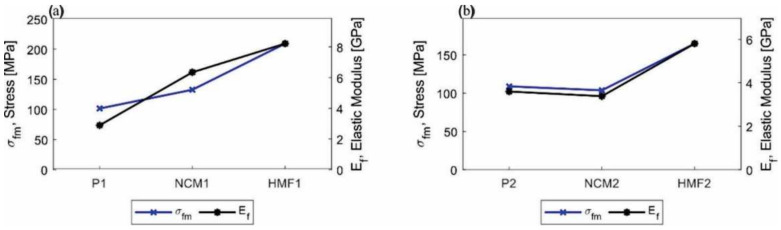
Graphic comparative summary of the maximum effort and elastic modulus in bending. (**a**) Test pieces made with configuration 1 (P1, NCM1, and HFM1) and (**b**) test pieces made with configuration 2 (P2, NCM2, and HFM2).

The results of the bending tests are shown in Figure 6. For configuration 1 (P1, NCM1, HMF1), a noticeable increase in mechanical behavior in this state of load is observed. When comparing the NCM1 nanocomposite material with the P1 base material, there is an increase in the elastic modulus (E_f_) and in the maximum bending force (σ_fm_) of 119% and 31%, respectively (See Figure 7a). We see an increase in the slope of the stress–curve deformation. This improvement is attributed to the physical or mechanical interaction of the NPs and the epoxy matrix increasing the surface area benefiting the performance of the nano-reinforced material; however, it indicates that it is more fragile and loses ductility [45].

On the other hand, the HMF1 material shows an increase in the elastic flexion modulus (E_f_) of 184% and the maximum bending force (σ_fm_) of 106% compared to the base material P1. As in the previous case when observing Figure 7b, an increase in the slope of the stress–deformation curve is observed, but the increase in mechanical properties is directly related to the functionalization of the nanoparticles of ZnO. The creation of covalent bonds increases cross-linking by decreasing the free volume and, in turn, forms a robust 3D structure between the resin, hardener, and ZnO-NPs-GPTMS. This reaffirms the importance of chemical interactions in the material: The absence considerably decreases all the properties of the base material. These results are consistent with A. Afzal [46], who showed that functionalized silicon nanoparticles mechanically strengthen the base material, increasing its resistance to stress by 60%, the Young’s modulus by 85% and the tensile strength by 56% and decreasing its malleability by 15%.

For configuration 2 (P2, NCM2, HMF2), although there is an increase in bending behavior, there is a difference in the NCM2 material. As seen in Figure 6, the effect of the NPs does not benefit the mechanical bending properties, decreasing the elastic modulus (E_f_) and the maximum bending effort (σ_fm_) at approximately 5%. While it was reported that the addition of NPs benefits the NCM1 material, there is also the possibility that the material may decrease its properties due to physical interaction, causing agglomeration and resulting in weak points in the matrix [47]. When observing the behavior of the HMF2 material there is an improvement in the elastic modulus (E_f_) and the maximum bending force (σ_fm_) of 61% and 51%, respectively. The functionalized nanoparticles anchored to the base material had the same effect as in the HMF1-type test pieces. The cross between the epoxy groups of the resin and the ZnO-NPs-GPTMS with the hardener diamines increased the bending resistance. This reaffirms the findings of A. Kausar et al. [48], who demonstrated that functionalized silica hybrids increased mechanical properties due to covalent chemical interactions.

A summary of the main properties evaluated is shown in Table 3 and Figure 7. When comparing the results obtained for each type of material, it is noted that type 2 material benefits much less from the addition of nanoparticles with and without functionalization in this state of charge, as can be seen in Figure 7. This phenomenon is associated with the type of epoxy matrix used, since it has been shown that matrices with a low elastic modulus (material P1 E_f = 2.89 GPa) that are nano-reinforced show a greater increase in their mechanical properties, whereas for matrices with a high elastic modulus (material P2 = 3.59 GPa) the effect can be null or negative [49,50].

**Figure 8:**
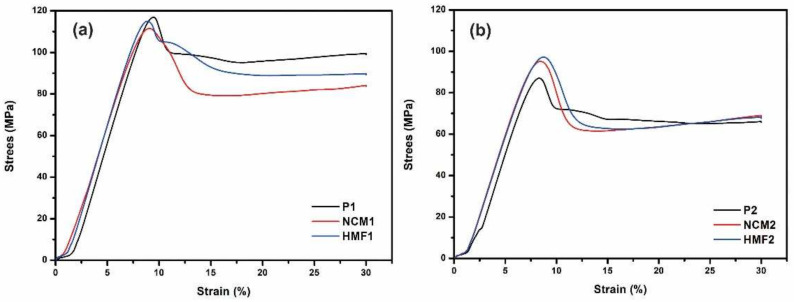
Graphic of the compression resistance for (**a**) test pieces made with cured resin 1 (P1), nanocomposite made with resin 1 and ZnO-NPs without functionalization (NCM1), and hybrid material (HMF1) and (**b**) test pieces made from cured resin 2 (P2), nanocomposite made from non-functioning resin 2, and ZnO-NPs (NCM2) and hybrid material (HMF2).

The results of the mechanical compression tests are shown in Figure 8. The test pieces produced in configuration 1 (P1, NCM1, HMF1) show a decrease in their mechanical compression properties in the order of 5% or less. When comparing the NCM1 nanocomposite material with the P1 polymer, no clear change in the compression modulus (E_c_) is observed, while the maximum compression stress (σ_cm_) decreases by 6%. The same phenomenon is observed in the test pieces of the hybrid material HMF1, where the elastic modulus in compression (E_c_) and maximum compression stress (σ_cm_) decreases by 0.4% and 4%, respectively. The explanation for these results is related to the dispersion of the nanoparticles within the base material: The density of the functionalized ZnO caused it to decant to the bottom of the test pieces, a phenomenon similar to that which occurred in bending. Figure S3 shows this defect. On the other hand, the values of the parameters that imply ductility are shown according to the expected effect of covalent bonds between the matrix and the nanoparticles, being less than the original material.

**Figure 9:**
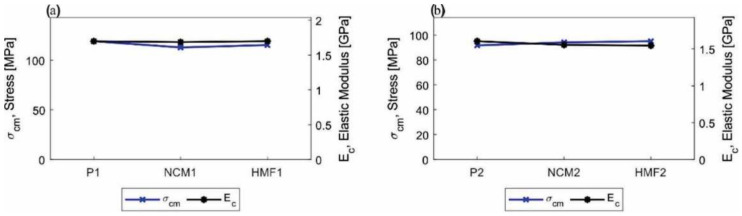
Graphic comparative summary of the maximum effort and elastic modulus in compression. (**a**) Specimens manufactured with configuration 1 (P1, NCM1, and HFM1) and (**b**) specimens manufactured with configuration 2 (P2, NCM2, and HFM2).

For materials with configuration 2, the trend is similar to that observed in configuration 1 materials, showing a decrease in mechanical compression properties in the order of 4% or less. It is interesting to note that for the NCM2 and HMF2 configurations, there is a reduction in the elastic modulus in compression (E_c_) of 3% and 4%, respectively, while the maximum compression stress (σ_cm_) increases slightly in the same proportion. The mechanical properties evaluated in the compression test are shown in detail in Table 4 and Figure 9. When comparing the results obtained for each type of material and configuration, it is observed that there is no great benefit when adding nanoparticles with and without functionalization in this state of charge, contrary to the result observed in the bending tests. This phenomenon is in line with the literature [51,52], since in the state of compression load, the addition of NPs only has a significant effect for high deformation states and in cyclic or fatigue states of load.

### 3.5. Thermal Properties

**Figure 10:**
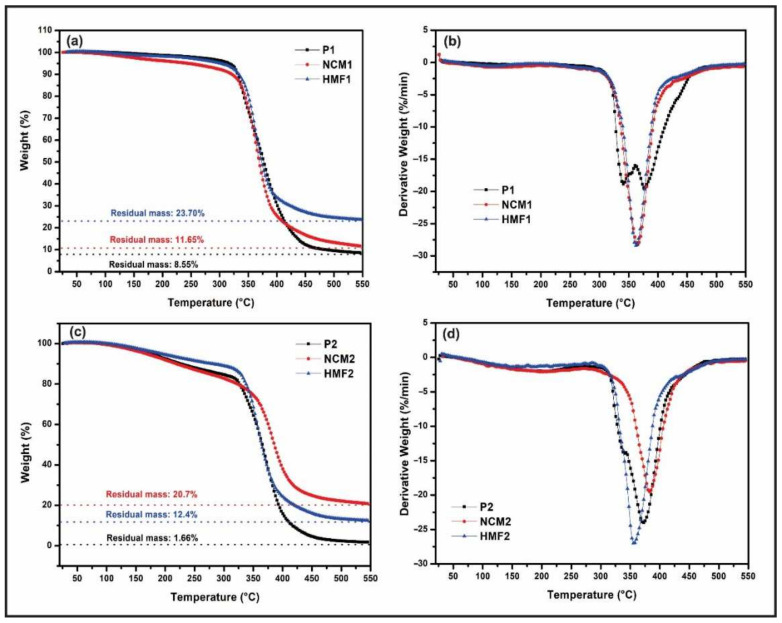
TGA and DTG analysis of the obtained materials. (**a**,**b**) Comparison of TGA and DTG analyses for P1, NCM1, and HMF1, respectively. (**c**,**d**) Comparison of TGA and DTG analyses for P2, NCM2, and HMF2, respectively.

In Figure 10a,b, the materials produced from the R1 + H1 mixture are shown in TGA and DTG analysis. The amount of residual mass for polymer P1 is 8.55%, 11.65% for hybrid material HMF1, and 23.7% for nanocomposite NCM1. Before 300 °C, a small degradation occurs in the three materials, due to the removal of water from the ZnO-NPs [53]. In the range between 300 and 400 °C a progressive and pronounced degradation begins, mainly of the carbon element in the base of the epoxy. In the case of hybrid material, the degradation of the silane anchored to the nanoparticles is added [54]; however, at these temperatures the nanoparticles do not degrade, giving a small increase in the high temperature resistance to the material as a whole. The greater amount of residual mass of HMF1 and NCM1 composite material compared to P1 polymer indicates that inorganic reinforcement gives degradation resistance to the base material.

Conducting the same analysis above for materials made from R2 and H2, it can be seen that the amount of residual mass for polymer P2 is 1.66%, 20.7% for hybrid material HMF2, and 12.4% for composite material NCM2 (Figure 10c,d). As for the temperature cycle, at the beginning, and before 300 °C, there is a small degradation in the three materials. This relates to the removal of water absorbed by ZnO-NPs-GPMTSs and ZnO-NPs in their synthesis and functionalization processes. At 350 °C, a progressive and noticeable degradation begins. In the case of polymer P2, the degradation of the carbon elements belonging to the epoxy resin begins and stops at approximately 400 °C. In the HMF2 material, the degradation of the anchored silane is initiated and maintained up to 400 °C. For NCM2 there is only degradation of the carbon chains since they do not have functionalized nanoparticles. The presence of ZnO-NPs gives HMF2 and NCM2 greater resistance to high temperatures compared to P2 polymer. This explains the greater amount of degraded mass of the latter. Meanwhile, the NCM2 has higher resistance to high temperatures than P2 and HMF2, which is due to the chemical structure of the R2.

### 3.6. Morphological Characterization

**Figure 11:**
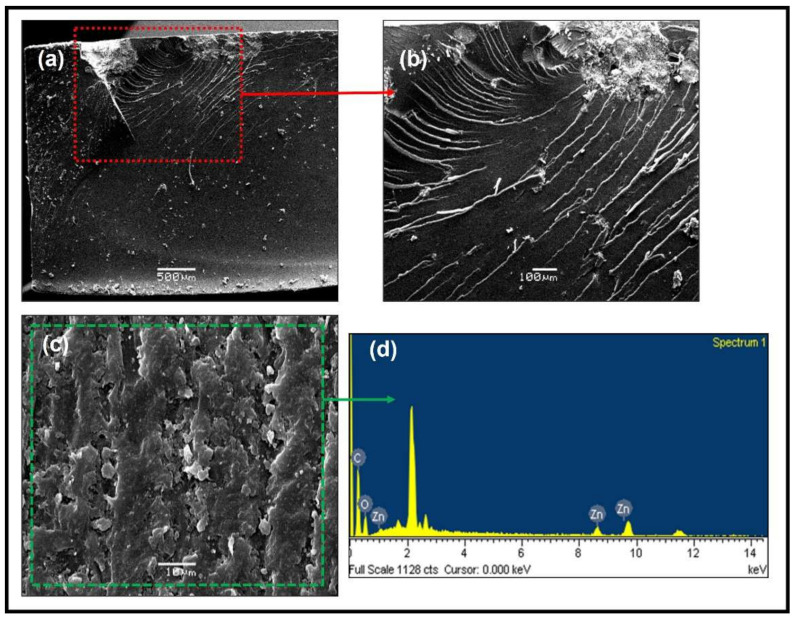
(**a**,**b**) SEM analysis of the fracture of hybrid materials (HMF1), (**c**,**d**) SEM microscopy of the analyzed surface and EDS spectrum.

In the micrographs of Figure 11a,b, one of the fracture zones of the HMF1 test piece can be seen. They clearly show the beginning of the fracture, which is typical of a fragile rupture due to the submission to high efforts. Meanwhile, the surface area is perceived to be irregular. EDS analysis of Figure 11d shows the presence of carbon, zinc, and oxygen. The carbon comes mainly from the epoxy resin, while the other two come directly from the ZnO-NPs anchored in the epoxy matrix. The results show a homogeneous distribution of carbon within the polymer matrix. Similar results showed the elements oxygen and zinc (Figure S4).

**Figure 12:**
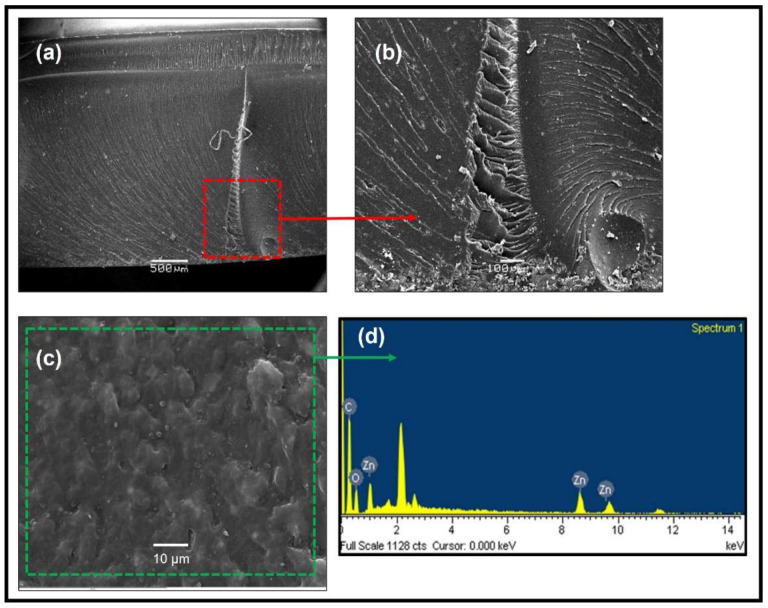
(**a**,**b**) SEM analysis of the fracture of hybrid materials (HMF2), (**c**,**d**) SEM microscopy of the analyzed surface and EDS spectrum.

Regarding HMF2, the lines showing the direction of the beginning of the fracture can be observed in the lower right area of the test piece (Figure 12a,b). It can be determined that the fracture began around the cavity present in that area, and the morphology of the fracture is typical of a fragile fracture. In addition, micrographs show a surface not free of small porosities, typical of the manufacturing process (mixing process). SEM mapping analysis for micrography (Figure S5) demonstrates the homogeneity of the carbon and oxygen elements within the matrix in that area and an inhomogeneity of the zinc element from the nanoparticles, which is related to the dispersion problem that arose in the manufacturing of the test pieces.

## 4. Conclusions

Nanoparticles of ZnO with hexagonal structure type wurtzite were obtained and verified using XRD analysis. These nanoparticles have a polyhedral morphology and are distributed heterogeneously, with an average size between 50 and 100 nm, results obtained from TEM analysis. In the FT-IR spectroscopy tests, the correct formation of covalent bonds resulting from functionalization with silane agents was verified. The Si-O-Si, C-H, and Si-O bands of the ZnO-NP functionalization reaction with the silane agent GPTMS were found. The increase in mechanical bending properties in the two final hybrid materials compared to the corresponding polymers was verified, achieving an average increase of 67% with a moderate decrease in ductility. In the case of compressive strength, they showed mixed results, maintaining the properties. Finally, with respect to thermal properties, it is concluded that inorganic reinforcement gives resistance to degradation to the base material, giving a greater resistance to high temperatures.

## Figures and Tables

**Table 1:** Composition of the resins and hardeners used.

(R1) Resine (L20) (Chemical Composition)	(H1) Hardener (EPH 573) (Chemical Composition)
BisPhenol-A-Epichlorhydrin (70%) Bisphenol-F-Epichlorhydrin (20%) Epoxidized molecule that gives flexibility to the thermoset polymer as: 1,6-Bis(2,3-Epoxy-propoxy)hexane (10%)	Diethylenetriamine m-phenylene-bis(methylamine) (1%)
**(R2) Resine Bifenol F (Chemical Composition)**	**(H2) Hardener (HT2) (Chemical Composition)**
Bisphenol-F-(epichlorhydrin) (70%) Bisphenol-A-(epichlorhydrin) (20%) 1,6-Bis(2,3-Epoxy-propoxy)hexane (10%)	3 aminomethyl-3,5,5-trimethylcyclohexylamine
**Chemical Structures**	
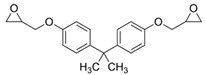	BisPhenol-A-Epichlorhydrin
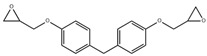	Bisphenol-F-Epichlorhydrin
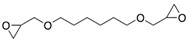	1,6-Bis(2,3-Epoxy-propoxy)hexane
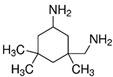	3 aminomethyl-3,5,5-trimethylcyclohexylamine
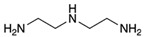	Diethylenetriamine
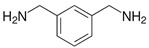	m-phenylene-bis(methylamine)

**Table 2:** Mixing relationships between resin, hardener, nanoparticles, and functionalized nanoparticles, for obtaining cured resins, nanocomposites, and hybrid materials.

Samples	Mixtures	Relation	Resin (g)	Hardener (g)	ZnO-NPs (g)	ZnO-NPs-GPTMS (g)
P1	R1 + E1	4:1	8	2		
HM1-G	(ZnO-NPs-GPTMS) + E1	4:1		1		4
NCM1	R1 + E1 + (ZnO-NPs)	(4:1) + (5% p/p)	16	4	1.05	
HMF1	R1 + E1 + (ZnO-NPs-GPTMS)	(4:1) + (5% p/p)	16	4		1.05
P2	R2 + E2	2:1	13.3	6.6		
HM2-G	(ZnO-NPs-GPTMS) + E2	2:1		2		4
NCM2	R2 + E2 + (ZnO-NPs)	(2:1) + (5% p/p)	13.3	6.6	1.05	
HMF2	R2 + E2 + (ZnO-NPs-GPTMS)	(2:1) + (5% p/p)	13.3	6.6		1.05

**Table 3:** Results of bending test analysis of the obtained materials.

Sample	E_f_	Improvement	ε_fm_	Improvement	σ_fm_	Improvement
	(GPa)	(%)	(%)	(%)	(MPa)	(%)
P1	2.89 ± 0.17	-	3.71 ± 0.21	-	101.56 ± 5.2	-
NCM1	6.35 ± 0.36	+119%	2.24 ± 0.78	−40%	132.75 ± 37.88	+31%
HMF1	8.22 ± 0.25	+184%	3.25 ± 0.19	−13%	209.36 ± 4.96	+106%
P2	3.59 ± 0.89	-	3.73 ± 0.39	-	108.68 ± 19.72	-
NCM2	3.38 ± 0.22	−6%	4.09 ± 0.57	+9%	103.61 ± 7.11	−5%
HMF2	5.81 ± 1.99	+61%	3.41 ± 0.58	−9%	164.61 ± 41.75	+51%

**Table 4:** Results of the compression test analysis.

Sample	E_c_	Improvement	ε_cm_	Improvement	σ_cm_	Improvement
	(GPa)	(%)	(%)	(%)	(MPa)	(%)
P1	1.69 ± 0.017	-	9.25 ± 0.35	-	119.88 ± 4.035	-
NCM1	1.69 ± 0.15	-	8.83 ± 0.21	−5%	112.83 ± 2.36	−6%
HMF1	1.68 ± 0.015	−0.6%	8.87 ± 0.19	−4%	115.23 ± 0.91	−4%
P2	1.61 ± 0.098	-	8.52 ± 1.078	-	91.72 ± 2.71	-
NCM2	1.56 ± 0.011	−3%	8.28 ± 0.16	−3%	94.11 ± 1.374	+3%
HMF2	1.55 ± 0.015	−4%	8.67 ± 0.19	+2%	95.04 ± 3.14	+4%

## Data Availability

Not applicable.

## Supplementary Materials

The following are available online at https://www.mdpi.com/article/10.3390/polym14081579/s1, Figure S1: (a) Molds for making specimens for bending and compression tests, respectively. (b) Polymer P1 specimens; (c) HMF1 final hybrid material specimens; (d) NCM1 nanocomposite material specimens; (e) Polymer P2 specimens; (f) HMF2 final hybrid material specimens; (g) NCM2 nanocomposite material specimens, Figure S2: Functionalization scheme proposed by Jaramillo et al. [37] and (b) proposed functionalization scheme with GPTMS, Figure S3: (a) Inhomogeneity of the ZnO-NP within the matrix (NCM1); (b) Surface defects of the specimen and small difference in thickness in specimen (P1), (c) Final hybrid material compression specimens MHF1, (d) Surface defects of the specimen (P2 and NCM2); (e) Differences in thickness in the specimen (MHF2), (f) Inhomogeneity of the ZnO-NP-funct-GPTMS within the matrix (HM2-G), Figure S4: (a) SEM-mapping analysis micrograph for final hybrid material HMF1 (b) Carbon element; (c) Oxygen element; (d) Element zinc, Figure S5: (a) SEM-mapping analysis micrograph for final hybrid material HMF2, (b) Carbon element; (c) Oxygen element; (d) Zinc element.
