# Supplementary material for: Hybrid Materials Based on Nanoparticles Functionalized with Alkylsilanes Covalently Anchored to Epoxy Matrices
Source: Polymers (Basel). 2022 Apr 13;14(8):1579. doi: 10.3390/polym14081579 (PMC9029025; doi:10.3390/polym14081579)
Supplement: Supplementary file 1 [file polymers-14-01579-s001.zip › polymers-1541938-supplementary.pdf]

polymers

MDPI

Supplementary Materials: Hybrid Materials Based on Nanoparticles Functionalized with Alkylsilanes Covalently Anchored to Epoxy Matrices

Alexis Salas, Andrés Felipe Jaramillo, Daniel Andrés Palacio, Andrés Díaz-Gómez, David Rojas, Carlos Medi-na, Eduardo Pérez-Tijerina, Francisco Solís-Pomar and Manuel Francisco Meléndrez

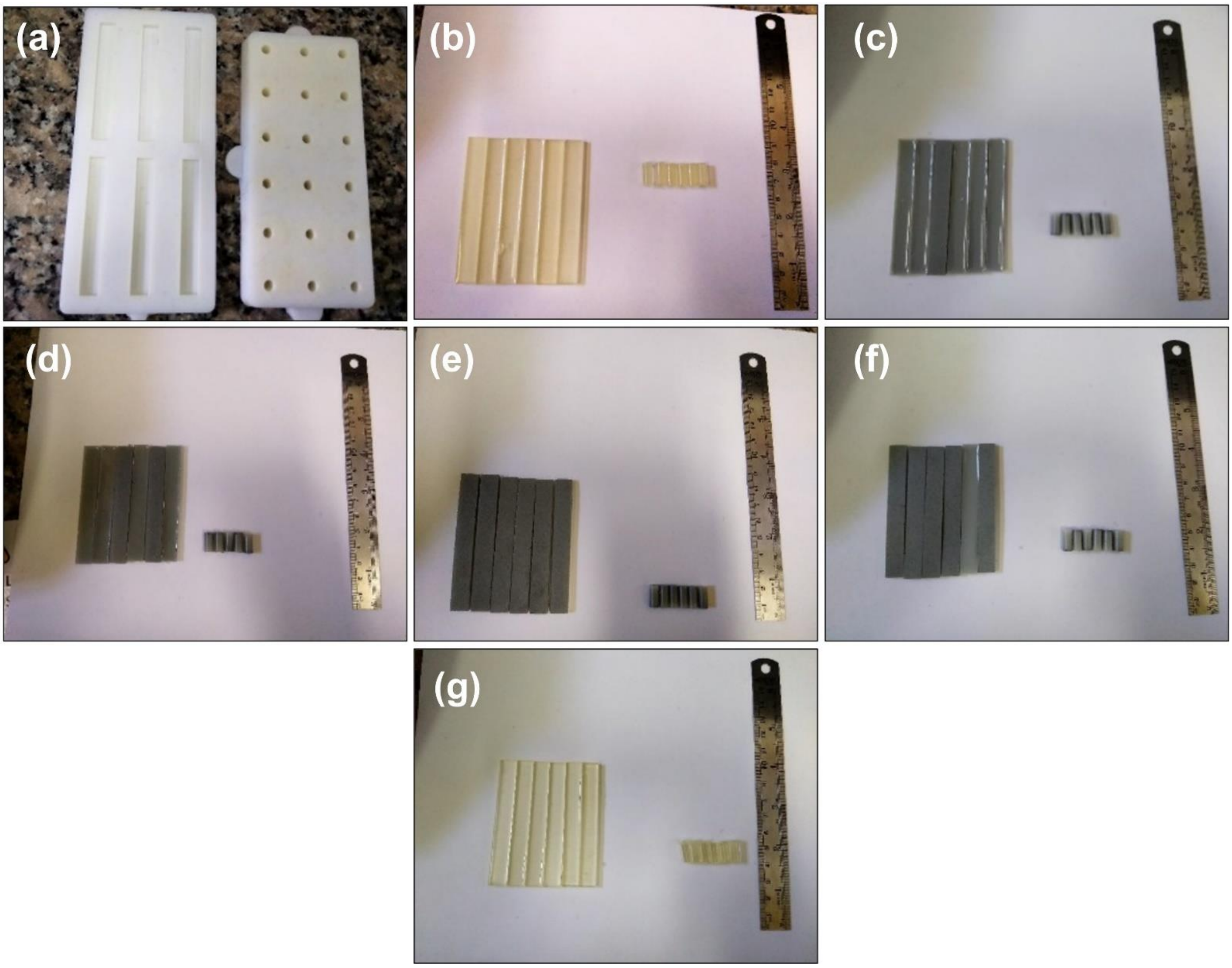

Figure S1. (**a**) Molds for making specimens for bending and compression tests, respectively. (**b**) Polymer P1 specimens; (**c**) HMF1 final hybrid material specimens; (**d**) NCM1 nanocomposite material specimens; (**e**) Polymer P2 specimens; (**f**) HMF2 final hybrid material specimens; (**g**) NCM2 nanocomposite material specimens.

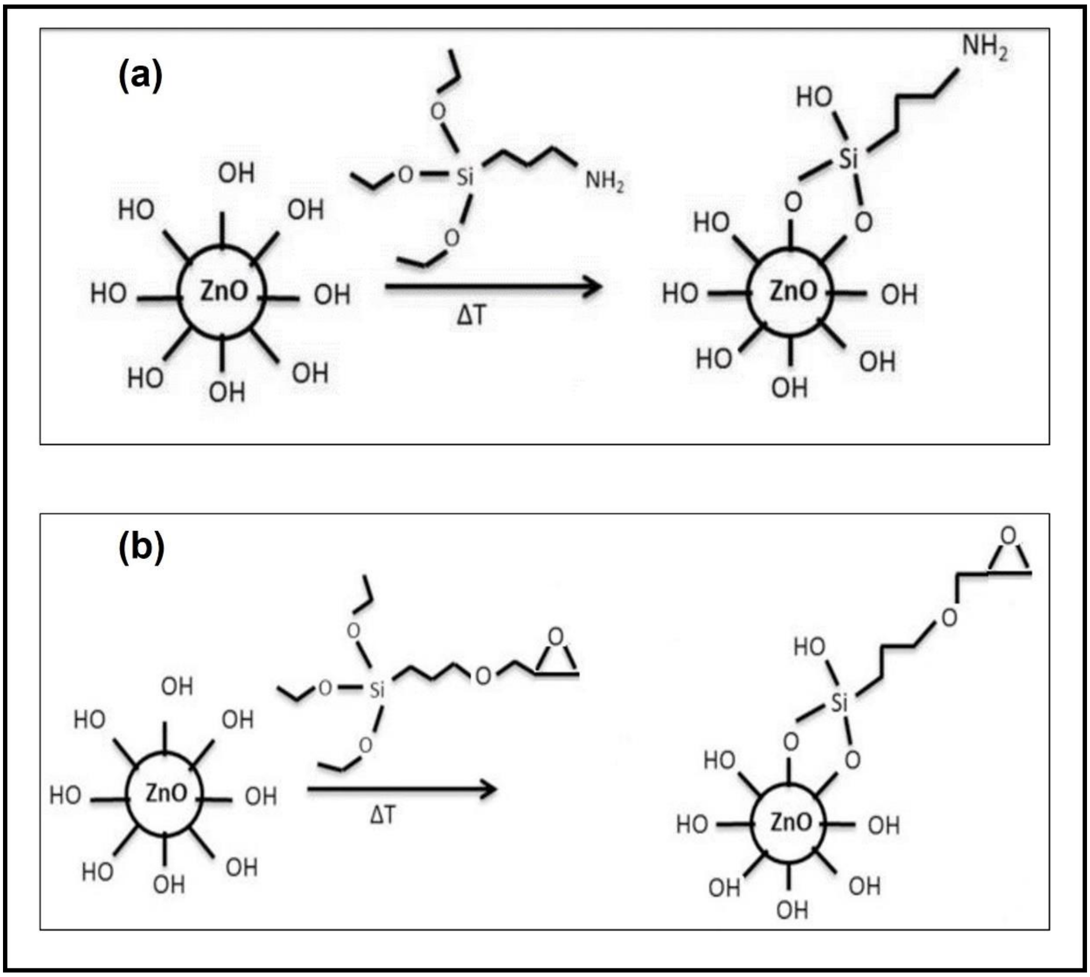

Figure S2. (**a**) Functionalization scheme proposed by Jaramillo et al. [37] and (**b**) proposed functionalization scheme with GPTMS.

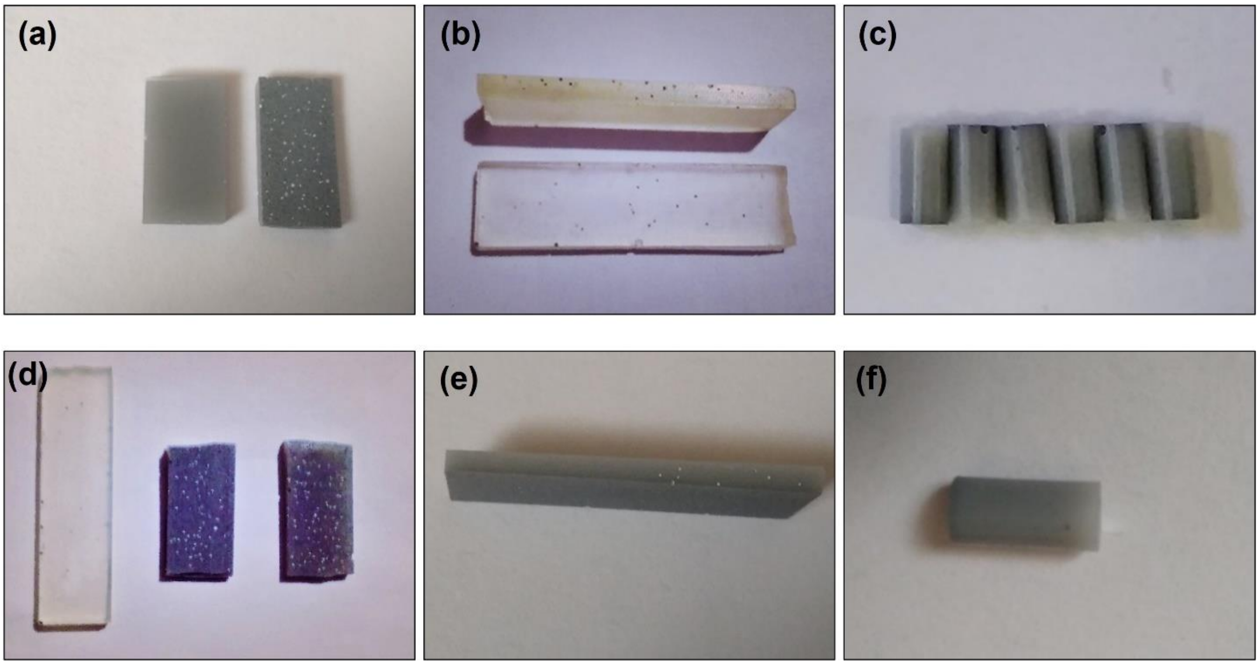

Figure S3. (**a**) Inhomogeneity of the ZnO-NP within the matrix (NCM1); (**b**) Surface defects of the specimen and small difference in thickness in specimen (P1), (**c**) Final hybrid material compression specimens MHF1, (**d**) Surface defects of the specimen (P2 and NCM2); (**e**) Differences in thickness in the specimen (MHF2), (**f**) Inhomogeneity of the ZnO-NP-funct-GPTMS within the matrix (HM2-G).

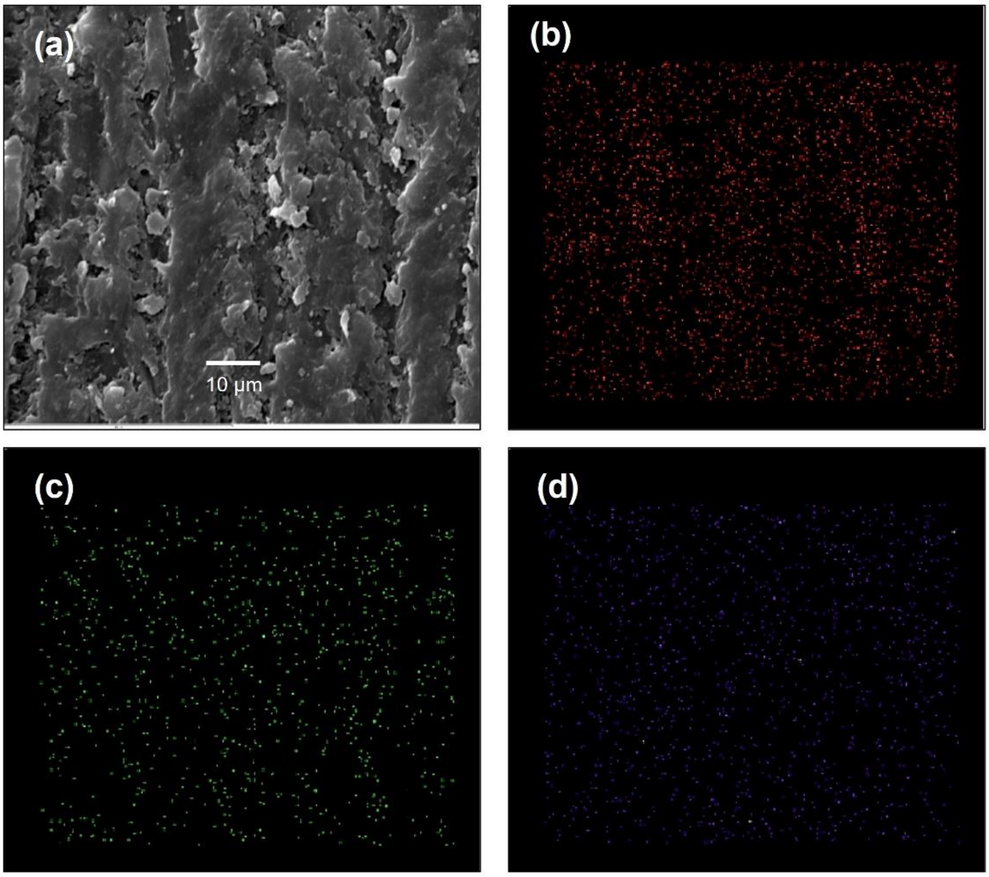

Figure S4. (**a**) SEM-mapping analysis micrograph for final hybrid material HMF1, (**b**) Carbon element; (**c**) Oxygen element; (**d**) Zinc element.

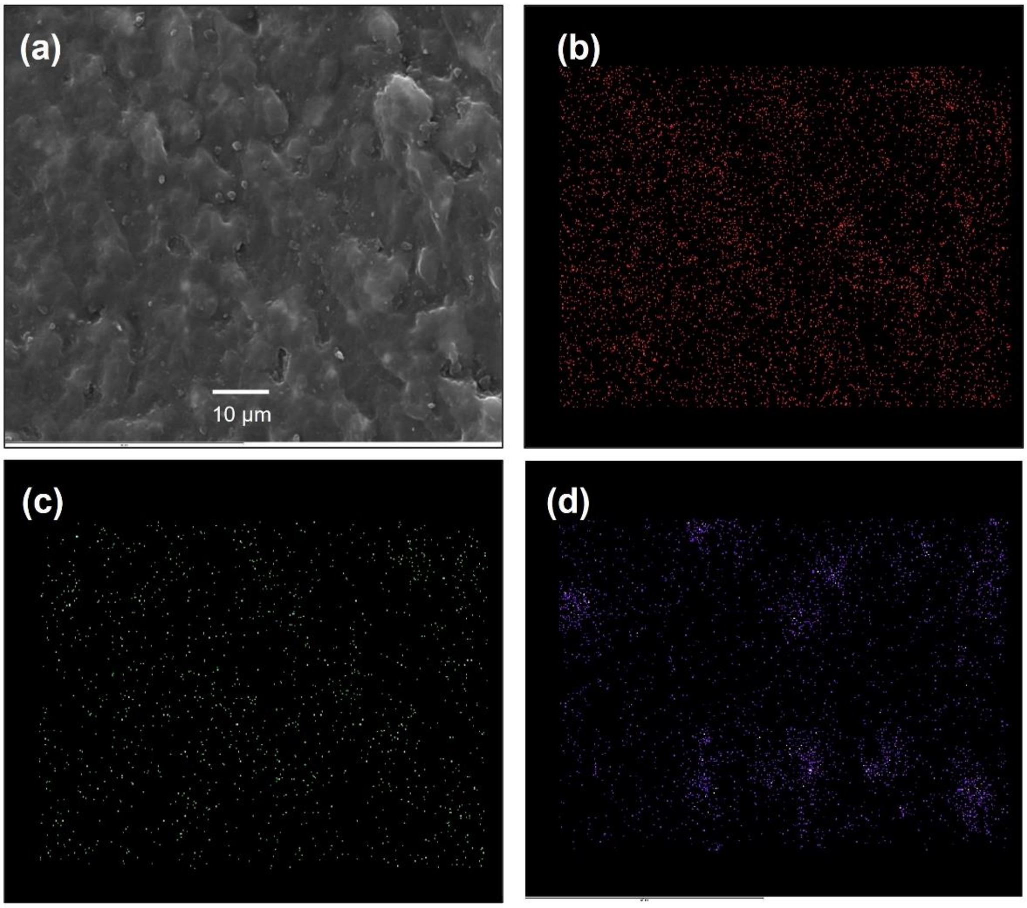

Figure S5. (**a**) SEM-mapping analysis micrograph for final hybrid material HMF2, (**b**) Carbon element; (**c**) Oxygen element; (**d**) Zinc element.
